# Mechanisms of Acupuncture Therapy for Simple Obesity: An Evidence-Based Review of Clinical and Animal Studies on Simple Obesity

**DOI:** 10.1155/2019/5796381

**Published:** 2019-02-03

**Authors:** Li-Hua Wang, Wei Huang, Dan Wei, De-Guang Ding, Yi-Ran Liu, Jia-Jie Wang, Zhong-Yu Zhou

**Affiliations:** ^1^College of Acupuncture and Orthopedics, Hubei University of Chinese Medicine/Hubei Provincial Collaborative Innovation Center of Preventive Treatment by Acupuncture and Moxibustion, Wuhan, China; ^2^Department of Acupuncture, Hubei Provincial Hospital of Traditional Chinese Medicine, Wuhan, China

## Abstract

Simple obesity is a worldwide epidemic associated with rapidly growing morbidity and mortality which imposes an enormous burden on individual and public health. As a part of Traditional Chinese Medicine (TCM), acupuncture has shown the positive efficacy in the management of simple obesity. In this article, we comprehensively review the clinical and animal studies that demonstrated the potential mechanisms of acupuncture treatment for simple obesity. Clinical studies suggested that acupuncture regulates endocrine system, promotes digestion, attenuates oxidative stress, and modulates relevant molecules of metabolism in patients of simple obesity. Evidence from laboratory indicated that acupuncture regulates lipid metabolism, modulates inflammatory responses, and promotes white adipose tissue browning. Acupuncture also suppresses appetite through regulating appetite regulatory hormones and the downstream signaling pathway. The evidence from clinical and animal studies indicates that acupuncture induces multifaceted regulation through complex mechanisms and moreover a single factor may not be enough to explain the beneficial effects against simple obesity.

## 1. Introduction

Simple obesity is a worldwide epidemic associated with rapidly growing morbidity and mortality which imposes an enormous burden on individual and public health. The expansion of fat mass, adipocyte size increase, and to a lesser extent cell proliferation (hyperplasia) are important features of simple obesity. It is generally believed that simple obesity is the second leading form of metabolic disorders after cardiovascular disease, with the latest projections estimating that there will be 2.16 billion overweight and 1.12 billion obese individuals globally by 2030 [1]. Nowadays, a number of studies have illustrated that obese people are faced with significantly increased risk of type 2 diabetes mellitus, cardiovascular disease, polycystic ovary syndrome, and a number of cancers in addition to impaired quality of life and social stigmatization [2–4]. In simple obesity and its complications, a plethora of aetiological mechanisms have been implicated which included unbalanced or disproportionate energy expenditure and food intake and a complex interplay between environmental and genetic factors that affect metabolic regulation, neurohormonal and haemodynamic, resulting in inflammation, lipotoxicity, apoptosis, oxidative stress, and more recently alterations (suppression or enhancement) in autophagy, a cardinal process for the regulation of energy homeostasis and cellular metabolism [5–8]. All the pathological changes occur throughout multiple functional systems of body and contribute to simple obesity. On the other hand, no definitive treatment for simple obesity has been explored currently. Treatment with pharmacological agents or nonpharmacological agents such as behavioural therapy and lifestyle modifications has not reached satisfactory results [9–11].

As an important technique of complementary and alternative medicine in the field of TCM, acupuncture has been utilized for both disease prevention and therapy for several thousands of years in China. The therapeutic effects of acupuncture are achieved via multiple pathways, including anti-inflammation effects, antiapoptotic effects, and neurotrophic effects [12–15]. According to the basic theory of TCM, the pathogenesis of simple obesity is mainly considered to be blood vessel obstruction by Qi deficiency and phlegm dampness stagnation, which might be caused by various internal and external factors such as stasis of liver qi, deficiency of spleen, or overintake of greasy flavour. The vital viscera associated with obesity in Chinese medicine refer to spleen, liver, kidney, and the Sanjiao (triple energizer). Based on the foundation of TCM theory, the therapeutic principle of simple obesity is invigorating spleen to remove dampness and relieving Qi stagnancy in liver to remove blood stasis [16, 17]. Not only ancient literature but also modern scientific evidence has shown the positive efficacy of acupuncture interventions in the management of simple obesity [18, 19].

Furthermore, numberous previous clinical and animal studies have been conducted to investigate the potential mechanisms of acupuncture on simple obesity. A deep insight into the endogenous mechanisms could provide opportunities to develop accurate strategies in the treatment of simple obesity. Crucially, the underlying molecular mechanisms have not yet been comprehensively reviewed. In this article, we review the possible mechanisms of acupuncture on simple obesity in clinical- and animal-based studies to provide guiding references for future research directions.

## 2. Evidence from Clinical Studies

### 2.1. Acupuncture Regulated Endocrine System in Patients of Simple Obesity

Over the recent years, endocrine disorders have been widely recognized as an important pathogenic factor of simple obesity. Related clinical studies indicated that obese patients without any treatments showed higher apparent endocrine disorder and complex endocrine metabolic mechanism [20]. A study based on 64 obese adult patients illustrated the relationship between the level of plasma nesfatin-1 and the metabolic and anthropometric parameters in obese adults and the features of the regulatory mechanism of acupuncture about endocrine system [21]. Nesfatin-1, which is a new hypothalamus peptide derived from nucleobindin 2(NUCB2), is distributed in the appetite-associated hypothalamic nuclei, such as paraventricular nuclei, arcuate nuclei, supraoptic nuclei, and lateral hypothalamic areas [21]. Moreover, systemically regulated nesfatin-1 inhibits body weight growth and appetite in rodents, demonstrating that this is an anorexigenic hormone [22]. Acupuncture significantly inhibited body weight, fat mass, body mass index (BMI), and waist circumferences (WC) and increased the nesfatin-1 level of plasma in obese patients after being performed for 30 min. Electrical stimulation was given by a pulse generator using 0.05 ms pulse width at 2 Hz frequency at 3 V with bidirectional square waves. Zhongwan (CV12) was connected to Tianshu (ST25) on one side, Guanyuan (CV4) was connected to ST25 on the other side, Zusanli (ST36) was connected to Sanyinjiao (SP6), and Fenlong (ST40) was connected to Yinlingquan (SP9) on the same side. It has also been supported that the nesfatin-1 level of plasma was negatively correlated with WC, BMI, and fat mass, which proved a negative association between metabolic parameters and nesfatin-1 level [21]. Besides, obese patients demonstrated significantly attenuated plasma ghrelin levels, known to cause weight loss and inhibited appetite, so that a reduction of body weight could raise plasma ghrelin levels in return. A study in obese subjects suggested that acupuncture could effectively reduce weight and partly restore the physiological function of ghrelin after the normal function was impaired [23]. A randomised controlled study that applied acupuncture treatment to obese women showed a decrease in leptin levels and a significant increase in ghrelin after 6 weeks of treatment [24]. The subjects were blinded and randomly divided into groups A and B. Group A received acupuncture treatment, and group B received sham acupuncture using placebo needles, twice each week for 6 weeks. Moreover, acupuncture enhanced the fasting blood Cholecystokinin (CCK) level, a hormone that is involved in feeding behavior through central and peripheral channels. CCK is said to be a neurotransmitter activating satiety signal by affecting the central nervous system after a meal, and there is a close relationship between CCK and ghrelin which starts the meal while CCK ends it [25]. Acupuncture also activated other endocrine-related cytokines such as insulin and epinephrine (Ad) but further studies are needed to confirm its role in the pathophysiology of simple obesity.

### 2.2. Acupuncture Promoted Digestion in Patients of Simple Obesity

Restoration of gastrointestinal function to the damaged digestive system has a vital impact on better weight management against simple obesity. Obese subjects treated with acupuncture showed decreased salivary amylase (S-Am), serum pepsinogen (SPG), and serum amylase (B-Am) after being performed for 20-30 min, and all the subjects received acupuncture treatment once every other day for 1 month, which indicated thay acupuncture therapy may be a useful method for inhibiting the function of hyperactive gastrointestinal digestion and absorption [26]. After acupuncture treatment, the active component such as 5-Hydroxytryptamine (5-HT) and Histamine (HB) in the gastrointestinal microcirculation decreased in obese patients, while prostaglandin E_2_(PGE_2_) increased on the effected side [27]. The local regulation mechanism may be attributed to the suppressed gastric acid secretion and colon contraction activity. Furthermore, a clinical trial by observing the changes of the surface electrogastrogram in obese patients showed that body acupuncture with auricular pressure for 1 month obviously delayed the increase of electrical amplitude in the stomach after the meal, indicating that acupuncture can delay the gastric emptying [28]. Body acupuncture was performed for 30 min, once every other day. And auricular pressure was given by once every five days and 1 month of therapy, for a period of treatment. Acupuncture also decreased the excretion rate of D-xylose, a natural pentose which can lead to the dysfunction of postprandial glucose levels via modulating gastrointestinal digestion as well as energy intake [26]. Moreover, acupuncture induced the feeling of satiety after meal blood-pressure-harmonic-variability responses, including decreases in food intake and increases in energy consumption on the obesity-affected side, while no significant changes were found on the contralateral side [29].

### 2.3. Acupuncture Attenuated Oxidative Stress

Oxidative stress, which is triggered by the imbalance between the clearance and generation of oxygen species (ROS), is a significant mechanism underlying obesity [30]. The overproduction of ROS usually may cause damage to proteins, lipids, and nucleic acids [31]. Simultaneously, antioxidants such as catalase, glutathione (GSH), superoxide dismutases (SOD), and glutathione peroxidase (GSH-Px) are decreased with ROS production [32]. Besides, excessive ROS could serve as the signalling molecules associated with inflammation and apoptosis as well. Therefore, increasing antioxidants or reducing oxidative stress might be a beneficial way in treating obesity [33].

In obese subjects, acupuncture treatment significantly decreased serum pro-oxidant antioxidant balance (PAB) values, which has been shown to be positively correlated to body weight and also increased in obese patients [34]. Eight acupuncture points on the abdomen, including Tianshu (ST-25) bilateral, Weidao (GB-28) bilateral, Zhongwan (REN-12), Shuifen (REN-9), Guanyuan (REN-4), Sanyinjiao (SP-6), and additional points including Quchi (LI-11) and Fenlong (ST-40) for excess mood (patients with higher energy) and Qihai (REN-6) and Yinlingqau (SP-9) for deficiency mood (patients with lower energy) on both lower legs, were selected. The needles in the abdomen were applied with electricity for 30–40* *Hz, dense-disperse wave. Each acupuncture treatment lasted for 20* *min. All subjects were asked to receive two treatment sessions per week for a total of 6 weeks. In previous studies, PAB values have been demonstrated to be elevated in patients with established coronary artery disease (CAD) and acute coronary syndrome (ACS)[35]. Furthermore, it has been indicated that PAB may be a potential cardiovascular risk predictor [36]. There was a significant association between weight and PAB values when the correlation was determined for all subjects, which indicated that the high levels of PAB values in obese patients without obvious symptoms/signs of cardiovascular disease might be related to a heightened state of oxidative stress associated with simple obesity [37].

Under oxidative stress circumstances, the overgeneration of ROS breaks the balance of thiol-redox environment and ultimately results in the disorder of biological activities in proteins. The thioredoxin system, which is comprised of thioredoxin reductase (TrxR), thioredoxin (Trx), and NADPH, is a major cellular antioxidant and thiol-reducing system [37]. Acupuncture could induce a series of responses among proteins associated with oxidative stress in obese subjects [38], but it is necessary to further discuss whether a cross-talk existed among different antioxidants about obesity regulated by acupuncture treatment.

### 2.4. Acupuncture Modulated Relevant Molecules of Metabolism

In obese patients, serum substances such as sugar, lipid, and obesity-related proteins are significantly higher, implying that the serum substances could be the representative biomarkers for the diagnosis of obesity or serve as surrogate markers. A recent analysis showed that acupuncture modulated the secretion of multiple serum lipids in obese patients. Acupuncture downregulated the level of serum triglyceride (TG), total cholesterol l(TC), and low-density lipoprotein cholesterol(LDL-C) and upregulated the level of serum high-density lipoprotein cholesterol(HDL-C), which could contribute to the removal of excess blood lipid[39]. All the obese subjects were asked to receive a total of 10–12 acupuncture sessions for 6 weeks; each acupuncture session lasted for 30 min. At the time of the first acupuncture session, all participants will be given a “dietary and exercise manual.” However, it was reported in other study that the level of TG, TC, LDL-C was significantly decreased but no changes in HDL-C were found after acupuncture treatment when compared with control groups [40]. EA was performed using the ear points, such as Sanjiao (Hungry) and Shen Men (Stomach) and several body acupoints for 30 min, once daily, for 20 days, whereas patients on diet restriction had a 1425(kcal) diet program, which consisted of 1425 Kcal daily for 20 days. In addition, a similar pattern of changes in TG, TC, LDL-C, and HDL-C has been reported as the previous study following acupuncture [41, 42], This may be explained by the difference of acupoints selection. It has also been suggested that the changes above associated with lipid metabolism may be caused by the increase of the betaendorphin levels in serum [40]. The decrease of the glucose level is associated with the formation of glycogen and the aggregation of blood insulin. Acupuncture significantly decreased the glucose level with a diet restriction that entailed a 1450 kilocalorie (kcal) diet program once a day for 30 min over 20 days, indicating that acupuncture could regulate the circulation of blood glucose in obese patients [41].

Circulating Serum C-reactive protein (CRP) is involved in the genesis and development of obesity as well. As a typical feature of systemic chronic inflammation, CRP is positively associated with parameters of adiposity such as WC and BMI as demonstrated by two large controlled clinical trials [42, 43]. Interestingly, the analysis showed that no changes of hs-CRP levels were found after treatment when compared with control, implying the notion that hs-CRP changed independent of the effects of acupuncture [44] and a mass of studies are still needed to prove it. Acupuncture also reduced the levels of serum insulin and other cytokines, such as leptin and C-Peptide in obese patients [41].

## 3. Evidence from Animal Studies

### 3.1. Acupoints Selection

**Figure 1:**
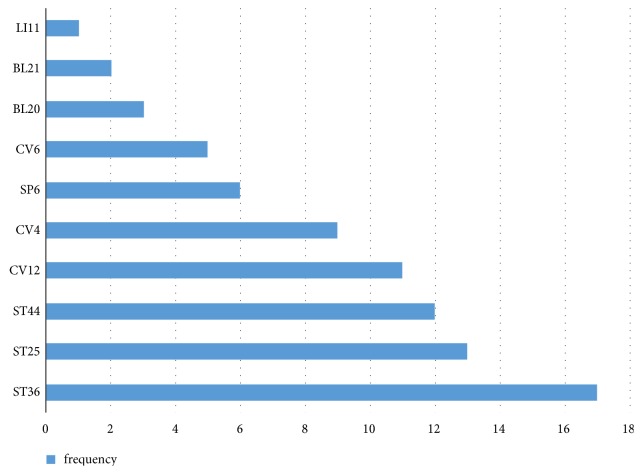
The frequent acupoints and their frequencies.

**Figure 2:**
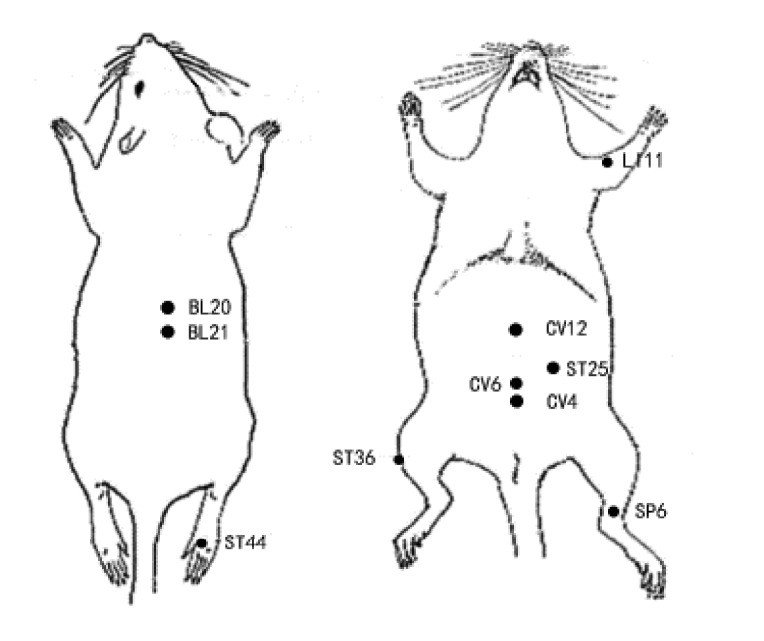
The location of frequently chosen acupoints on rat for treating simple obesity.

Current studies show that although acupoints selection varies significantly, certain acupoints are used more frequently than others (Figure 1). To be specific, the following acupoints are more likely to be used on animal models of simple obesity by researchers: Zusanli (ST36), Tianshu (ST25), Neiting (ST44), Zhongwan (CV12), Guanyuan (CV4), Sanyinjiao (SP6), and Qihai (CV6). A schematic location of acupoints mentioned in Figure 1 is shown in Figure 2.

### 3.2. Mechanisms of Acupuncture on Simple Obesity

#### 3.2.1. Acupuncture Regulated Lipid Metabolism

It is well known that lipid metabolism is a complicated physiological process, which involves lipid synthesis, intake, transportation, etc. Lipids are mainly derived from the internal synthesis of acetyl coenzyme A and food intake. The disorder of lipid metabolism is characterized by abnormal lipid secretion and the metabolites of lipid, which is caused by congenital or acquired factors, distributed predominantly in plasma and other tissues as well [45]. Dyslipidemia, which upregulated the levels of serum lipid and lipoproteins in particular, has always been considered to be the common pathology of obesity and other complications directly or indirectly [46]. The key to maintain lipid homeostasis is whether excessive synthesis or uptake of lipids* in vivo* can be metabolized or transported to other places outside the cell membrane. Similarly, lipid efflux also involves a mass of proteins, including ApoE, ABCA1, ABCA1, Apolipoprotein (Apo) A-1, peroxisome proliferators-activated receptors (PPARs), and scavenger receptor class B type 1(SR-B1) [47].

In physiological conditions, adiponectin is exclusively secreted by adipocytes and activates adenosine monophosphate-activated protein kinase (AMPK). In turn, activation of AMPK inactivates acetyl-CoA carboxylase (ACC) by phosphorylation and blocking the transformation of acetyl-CoA into malonyl-CoA [48]. However, in obese conditions, increased adiposity leads to low plasma levels of adiponectin production, resulting in a decreased AMPK activity. The inactivation of AMPK reduces PPAR-*α* activity and the phosphorylation of ACC [49], inducing hyperglycemia [50]. Acupuncture not only led to a reduction in the weight of body, liver, and fat pad in obese rats, but also a decrease of TC, TG, fatty droplet accumulation, and serum concentrations of AST and ALT simultaneously. Furthermore, acupuncture increased CPT-1 expression and restored phosphorylation levels of ACC (Ser79) and AMPK (Thr172) that were inhibited by high fat food (HFD)[51].

The beneficial therapeutic effects of acupuncture treatment could attribute to the regulation on lipid metabolism. A study using Obese Zucker Diabetic Fatty Rats reported that repeated acupuncture at Zhongwan (CV12) and Guanyuan (CV4) acupoints caused an increase in adiponectin and a decrease in leptin [52]. Recently, another study also demonstrated that acupuncture lowered the weight of visceral white adipose tissue (WAT) and increased the levels of protein pPKA, pPKC, pERK and TRPV1 in DRG and SC [53]. TRPV1 are responsible for uptaking and releasing of lipid. A laboratory investigation showed the key role of TRPV1 mRNA in visceral adipose tissue by exploring the relationship between normal caloric intake and the visceral fat [54].

#### 3.2.2. Acupuncture Modulated Inflammatory Responses

Inflammatory responses, aimed at defending the body from various insults including tissue damage and infection and ultimately leading to the restoration of morphological and functional integrity of affected tissues, play a key role in the pathophysiological process of simple obesity[55, 56]. Typically, during acute inflammation, a mass of immunomodulatory molecules such as chemokines and cytokines are released from tissue-resident macrophages and mast cells which are triggered by the initial insult; these molecules provoked a rapid recruitment of neutrophils first and then lymphocytes and macrophages from circulation to the inflammation site [57]. The infiltrating cells then destroy the infectious agents and remove damaged cells, and finally the transition is performed via T- and B lymphocytes and antigen-presenting cells (APC) from innate to adaptive immunity [56, 57]. Simple obesity has been shown to be closely related to a different type of inflammation which is referred to as chronic low-grade inflammation, and it is characterized by a modest increase of proinflammatory factors in the circulatory system and the absence of clinical signs of inflammation (hence the term subclinical inflammation)[58].

Acupuncture reduced mRNA levels of several cytokines in adipose tissue, including interleukin-6(IL-6), monocyte chemotactic protein-1(MCP-1) and tumor necrosis factor-*α* (TNF-*α*) [59]. IL-6 and TNF-*α*, which are pro-inflammatory cytokines, have been confirmed to be elevated in obese subjects [60]. Many previous studies have indicated that IL-6 and TNF-*α* are involved in obesity-related diseases like atherosclerosis and insulin resistance [61]. Moreover, obesity could induce an increase of the serum concentrations of IL-6, TNF-*α*, and IL-1*β* in obese rats. EA treatment decreased the serum concentrations of IL-6, TNF-*α*, and IL-1*β* [62]. As a central regulator of various cellular genes in adipose tissue, the transcription factor NF-*κ*B involves various inflammatory responses and undergoes phosphorylation and then an ubiquitination-dependent degradation in response to the stimulation by factors such as I*κ*B and TNF-*α* [63, 64]. There is evidence that the NF-*κ*B signaling pathway could be an appropriate therapeutic target to defend chronic inflammation [65]. By inactivating this signaling pathway, EA exerted its beneficial effects on inflammation associated with obesity in the adipose tissue [33]. Acupuncture at CV12/CV4 acupoints also decreased adiponectin and increased serum leptin significantly [66], and repeated acupuncture in obese rats increased IL-10 and decreased serum TNF-a accordingly [67]. In summary, acupuncture has been shown to alter the balance of pro- and anti-inflammatory cytokines in obese rat models. Besides, another study suggested that acupuncture significantly decreased the TNF-*α* level in blood serum, and the decrease of serum TNF-*α* level by acupuncture treatment is consistent with previous reported studies [62, 68]. The decrease of TNF-*α* indicated that acupuncture was effective in reducing insulin resistance and in reducing the overall inflammatory state of the obese rats, which were characterized by adipocyte hypertrophy and hyperplasia [69], decreased levels of adiponectin [70], and increased numbers of activated macrophages in the white adipose tissue (WAT)[69].

Acupuncture also regulates other obesity-associated inflammatory cytokines. Interleukin-10 (IL-10) was originally synthesized within multiple organs which included spleen and is responsible for the inhibition of the synthesis of pro-inflammatory cytokines [71, 72]. A large number of IL-10 are generated from activated B-cells that mature in the marginal area of spleen [73]. Acupuncture could decrease pro-inflammatory cytokines by reducing the production of IL-10 [74]. In addition, the effects of acupuncture on inflammatory responses were related to multiple complicated systems, all of which are collectively responsible for weight loss via participating in the anti-inflammatory cytokines of formation, transmission, and reorganization.

#### 3.2.3. Acupuncture Suppressed Appetite through Regulating Appetite Regulatory Hormones and the Downstream Signaling Pathway

Appetite is known to be primarily dominated by a complex circuit of hypothalamic nuclei which is involved in series of satiety/appetite signals, action areas about messengers regulatory, and act sites. The hypothalamic arcuate nucleus (ARC), which is the major appetite regulatory site, receives input from central and peripheral (pancreas, adipocytes. and brain) sources [75]. The ARC consists of at least two populations of neurons with opposing functions on food intake: primarily lateral anorexigenic (pro-opiomelanocortin; POMC and cocaine- and amphetamine-regulated transcript; CART) and primarily medial orexigenic (neuropeptide Y; NPY and agouti-related protein; AgRP) neurons [76, 77]. In general, the hypothalamus affects feeding by integrating peripheral humoral signals that influence energy expenditure and food intake, with neural signals that come from the brainstem or even higher cortical centres. The key role of the hypothalamus in energy homeostasis was first suggested by classic lesioning experiments in rodents [78]; subsequent studies have shown the importance of hypothalamic nuclei in energy homeostasis, which included paraventricular nucleus (PVN), arcuate nucleus (ARC), dorsomedial nucleus (DMN), ventromedial nucleus (VMN), and lateral hypothalamic area (LHA) [79–81].

A previous study indicated that acupuncture up-regulated CART peptide expression in the ARC. As a hypothalamic satiety factor, CART neurons in the ARC contain a number of pro-opiomelanocortin (POMC) mRNA which plays a key role in the regulation of inhibiting appetite [82, 83]. It has been shown that intracerebroventricular (ICV) administration of CART inhibits food intake [82], whereas ICV injection of CART antibody increases food intake [82]. All the observations above indicated that both increased CART and POMC (*α*-MSH) expression could contribute to the anorexigenic effect of acupuncture. The ARC, which is a core hypothalamic nucleus in the regulation of appetite, is well established to integrate a mass of peripheral signals associated with food intake, such as insulin and leptinand ghrelin [84, 85]. Furthermore, the mTOR signaling pathway was involved in the regulation of acupuncture on energy metabolism. When the rats were fed, the activity of mTOR in the ARC of the hypothalamus increased, and the expression of target enzymes in the downstream also upregulated accordingly, which illuminates that the energy status of the body is associated with the mTOR activation, thereby clarifying the importance of hypothalamic mTOR in maintaining energy balance of the body [86]. By regulating the mTOR pathway, the stimulation of EA significantly reduced the level of the plasma leptin and simultaneously increased the serum leptin level of the hypothalamic in the obese rats [87]. Acupuncture elevated the binding of insulin and leptin with each receptor in the hypothalamus ARC as well so as to trigger a chain of biological reactions, namely, an inhibition of the appetite-gain activity of AgRP and NPY or an enhancement of the anorexic activity of CART and POMC, causing an increase in energy consumption and a decrease in food intake and thereby eventually achieving a weight loss goal [88].

The transient receptor potential vanilloid type-1(TRPV1), which is primarily expressed at the afferent neurons, also plays a key role in thermal, mechanical, chemical, and many other sensory transmission systems [89, 90]. Acupuncture stimulation was reported to increase the expression of TRPV1 that was endowed with nNOS immunostaining of subepidermal nerve fibers in the acupoints [91]. TRPV1 mediates sensory signal transduction [92], and TRPV1 has been suggested to contribute to body weight and feeding behavior signaling [93–95].

#### 3.2.4. Acupuncture Promoted White Adipose Tissue Browning

The adipose tissue is mainly composed of two types: white adipose tissue (WAT) and brown adipose tissue (BAT). The two tissues have different morphological distributions, gene expressions, and functions in the body. WAT stores energy and releases cytokines and hormones that regulate insulin resistance and metabolism, while BAT consumes energy mainly through mitochondrial uncoupling in nonshivering thermogenesis mediated by Uncoupling protein-1 (UCP-1), as an adaption process to cold exposure [96]. Recent studies about metabolically active BAT in adults [97–100] have highlighted BAT as a novel therapeutic target for obesity and other obesity-associated diseases, such as insulin resistance [101]. The activity of BAT is inversely related to BMI in humans [97], which implied the significant role of BAT during the development of obesity. In particular, the brown adipocyte-like cells distributed in WAT are shown to be generated by b3-adrenergic stimulation or cold exposure in rodents [102]. The transition of white to brown fat covers a series of processes in cell, including *β*-oxidation, lipolysis, mitochondria production, and increased UCP-1 expression. Importantly, the browning process and therefore thermogenesis are reported to be stimulated by a number of external factors like physical exercise, cold exposure, and medication [103]. Therefore, therapies aiming at activating thermogenesis in WAT might be a great strategy to prevent and treat simple obesity.

Recent studies indicated that acupuncture is notably effective in targeting WAT browning in obesity [104]. Acupuncture on Neiting (ST44) and Zusanli (ST36) acupoints remodeled WAT to BAT via inducing the expression of UCP-1 in obese rats, thus decreasing adipocyte size and promoting lipolysis in WAT. The overexpression of UCP-1 specifically seen in BAT is an important characteristic of the browning process. However, the expression of UCP-1 is regulated by a chain of molecular mechanisms, in which SirT1, Prdm16, and Ppar*γ* are involved. Acupuncture increased the expression of Ppar*γ* gene in WAT significantly. Specifically, SirT1-dependent deacetylation of Lys293 and Lys268 is required to recruit the BAT program co-activator Prdm16 to Ppar*γ*, resulting in the repression of visceral WAT genes and a selective induction of BAT genes associated with obesity-related diseases [105]. Furthermore, the acetylation of Ppar*γ* was obviously decreased after the acupuncture treatment, which indicated acupuncture might promote Ppar*γ* deacetylation so as to up-regulate the expression of UCP1 gene and then inducing the browning process. Moreover, other BAT markers such as Cox4il and Nrbf1 are also significantly elevated by acupuncture. Although, animal studies have found that acupuncture could promote weight loss and WAT browning, the detailed mechanisms at the cellular level by which the effectiveness exerted still stay unclear. Further studies in the clinical patients and animal models are of interest to make sure the significance of acupuncture on simple obesity in models.

## 4. Discussion and Consideration

**Figure 3:**
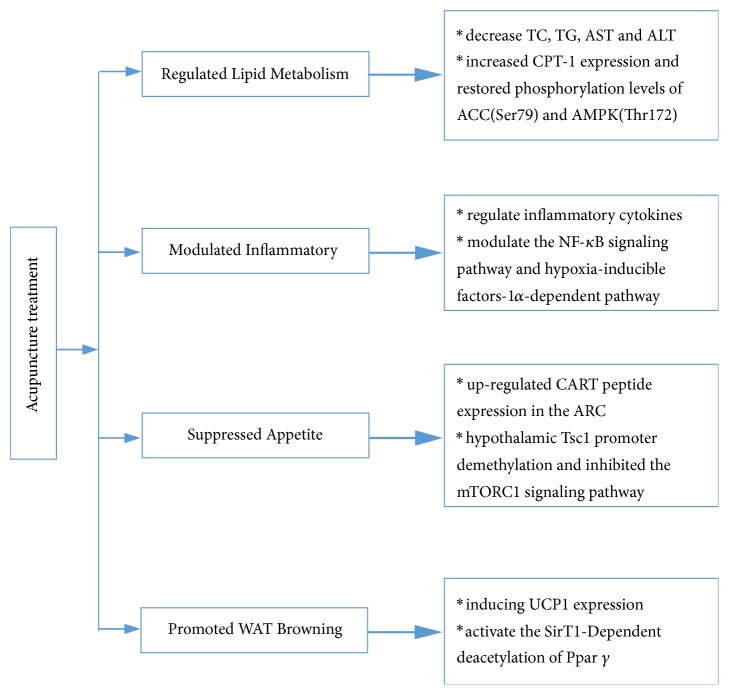
Effects of acupuncture on molecular and cellular events during the pathological process of simple obesity in animal models.

It is demonstrated that acupuncture applied at specific acupoints can contribute to the restoration of endocrine and metabolic disorders in subjects with simple obesity. The mechanisms behind the beneficial effects of acupuncture can be explained by molecular biology. In obese patients, acupuncture modulated cerebral nervous system and activated specific brain regions and relevant molecules. On the other hand, evidence from clinical and animal studies show that the therapeutic mechanisms of acupuncture nearly cover all the cellular and molecular events during the pathophysiological process of simple obesity (Figure 3). It seems that the effects of acupuncture are multitargets and the modulation induced by acupuncture on various pathways above ultimately paves the way for weight loss.

From the perspective of research, acupuncture may be regarded as a complementary and alternative therapy for simple obesity in which potential therapeutic targets can be explored and investigated. It requires more rigorous studies on how acupuncture exerts therapeutic effects by influencing relevant signaling pathways, specific brain regions, or molecules. However, it should be noticed that several issues with methodological weaknesses and small sample size are still needed to be discussed. Firstly, lots of literatures included in the current clinical study lack clear randomization methods and long-term follow-up. Secondly, the quality of some literatures chosen is not high and the number of literatures related to obesity is limited as well, so inadequate conclusions may potentially exist. In general, more and larger definite clinical and animal studies are necessary to advance our understanding of the basic mechanism and provide high quality evidence for future clinical application.

Based on findings to date, clinical studies about the mechanisms of acupuncture on simple obesity mainly focused on regulating endocrine system, promoting digestion and modulating relevant molecules of metabolism, while evidence from laboratory indicated that acupuncture regulates lipid metabolism, attenuates oxidative stress, modulates inflammatory responses and promotes white adipose tissue browning. Acupuncture also suppresses appetite through regulating appetite regulatory hormones and the downstream signaling pathway. These effects of acupuncture on simple obesity have been generally acknowledged. As early initiation of therapeutic forms is not always available in clinical practice owing to the limitations of therapeutic treatment window, acupuncture therapy may be a potential TCM method against damages induced by obesity.

To our knowledge, this study is the most comprehensive overview that systematically reviewed clinical and animal trials on acupuncture for patients with simple obesity. Despite the fact that acupuncture therapy demonstrated significant effects on obesity in the laboratory, several obstacles are included hampering the transition between animal and clinical research. Based on the accuracy and specificity of acupoint localization in acupuncture treatment and acupoint selection among over 300 acupoints of human body, the areas covered by the acupoints in animals are much less than that in human as animals involved were smaller. In the experimental acupuncture research, simulation of traditional acupoints on animals is the crucial step. The location of acupoints in experimental animals should be based on the simulation study of human body acupoints and combined with the animal experiments map. At the same time, the differences between animals and humans should be realized, and the simulation of human acupoints should not be simply projected to animals according to a certain proportion. So far, how many meridians located on rats specifically remains to be further discussion; this in itself is open to challenge and any results in relation to acupoints in rats are certainly open to criticism, which make it tenuous to be readily generalized to humans. What's more, acupoint prescriptions in clinical studies often contain ten or more acupoints with other medical interventions, while animal studies use only single or no more than five acupoints. From the research standpoint, clinical studies emphasize on the effectiveness of acupuncture which highlights standardization of acupuncture manipulation, while animal studies aim to explore potential molecular mechanisms underlying acupuncture manipulation. All of these differences make it difficult for animal studies to serve the clinical studies.

Appropriate acupuncture manipulation is an important factor that determines the results of acupuncture studies. The parameters including acupuncture models (MA or EA), acupoints, duration, and frequency in acupuncture manipulation may influence the efficacy of acupuncture. The mechanism underlying the therapeutic effects of acupuncture has been shown to be similar to therapies associated with activities, including dietary restriction, exercise, and enriched environment, many of which have been widely applied to simple obesity [106–108]. However, unlike these, acupuncture ultimately exerts therapeutic effects by stimulating specific acupoints which are correlated with the CNS and other systems in the body. To be exact, physical stimulation of peripheral nerves from a needle in a specific area can induce a series of neurophysiological responses in the CNS and other systems [106]. Therefore, in addition to selecting specific acupoints that promote the expression of nerve and humoral factors, illuminating the therapeutic targets in specific functional areas in which physiological activity or regulatory substance of signaling pathways is modulated by the stimulation of specific acupoint might be critical for developing a more efficient acupuncture therapy.

Although great advances have been made in the researches of acupuncture parameters, how these kinds of clinical and animal studies would be applied into clinical practices still remains a huge challenge. To overcome this flaw, we believe that the following problems should be paid attention to in future researches. Firstly, we should testify the changes of signaling pathways or relevant molecules in animal studies to comprehensively understand the mechanism behind acupuncture when using specific acupuncture parameter. Secondly, evaluate the optimal acupuncture regimen by making comparison of the therapeutic effects when we use different acupuncture parameters. Thirdly, we should justify whether the best acupuncture option acquired in animal studies could be applied to clinical studies. That is, the outcomes of animal studies should provide strong molecular biological evidence for acupuncture therapy and assist in screening out the optimal acupuncture therapeutic option for clinical.

In summary, acupuncture exerts beneficial effects via modulating a series of biological events in simple obesity. As an economical medical technology without adverse effects, acupuncture could be used in obese patients who cannot tolerate exercise or drug therapy and could also be used for lots of metabolic disorders along with other therapies. Further studies are still needed to explore the deeper mechanisms and their biological significance. In particular, future studies should focus on the potential mechanism of acupuncture on obesity-related complications such as hyperlipemia and polycystic ovarian syndrome, which is associated with metabolism dysfunction. Besides, more animal studies of acupuncture should be encouraged to be translated into clinical studies.
